# Mizaj assessment and data analysis methods in Amirkola health and aging project (AHAP cohort)

**DOI:** 10.22088/cjim.13.4.759

**Published:** 2022

**Authors:** Morteza Mojahedi, Roshanak Saghebi, Narjes Gorji, Reyhaneh Moeini, Seyed Reza Hosseini, Ali Bijani, Reza Ghadimi, Seyyed Ali Mozaffarpur, Hoda Shirafkan

**Affiliations:** 1 Traditional Medicine and History of Medical Sciences Research Center, Health Research Institute, Babol University of Medical Sciences, Babol, Iran; 2 Department of History of Medical Sciences, School of Persian Medicine, Babol University of Medical Sciences, Babol, Iran; 3 Social Determinants of Health Research Center, Health Research Institute, Babol University of Medical Sciences, Babol, Iran

**Keywords:** Geriatrics, Cohort studies, Medicine, Traditional, Complementary therapies, Temperament, Precision medicine

## Abstract

**Background:**

One of the principles of Persian medicine (PM) is the individualized approach that is presented with the concept of Mizaj. In this viewpoint, on the whole body, Mizaj is determined for every person based on 10 criteria, which is a result of the Mizaj of the main organs, including the brain, liver, and heart. There is no standard diagnostic tool for Mizaj assessment yet. The purpose of this study is to explain the method of Mizaj assessment and data analysis in the elderly in one of the biggest health and aging projects in Iran. The second phase of the Amirkola health and aging project (AHAP) evaluated more than 1,700 clinical and laboratory examinations of 2135 elderly people.

**Methods:**

In this study, a novel Mizaj assessment method in two phases is presented. In the first phase, 1541 elderly were assessed by a PM expert and typical diagnoses (the high confidence of expert’s proficiency) were determined. At the second phase, an expert panel including 5 PM experts evaluated the cases. The data of the elderly whose Mizaj agreed in the expert panel was used to assess its correlation with Mizaj. Also, the Mizaj of the main organs of these cases was evaluated this way.

**Conclusion:**

In the lack of valid and reliable questionnaires to assess the personalized viewpoint of PM, a new expert-based method has been introduced that can be used in similar studies. The result of the Mizaj assessment in this way will be used to obtain objective values for the Mizaj assessment.

Attention to complementary and traditional medicine has increased worldwide (1). One of these medical schools with a long history is Persian medicine (PM) (2). The foundations of PM are based on two perspectives: holistic viewpoints and individualized approach (3). The holistic view introduces the concept of administrative power of the body that results in the importance of lifestyle as the basis of any prevention and treatment. Person-centered views, with the word Mizaj, try to categorize all people in a range from warm to cold and also from wet to dry (4). In PM written references, 10 criteria for determining this diagnosis are stated (5, 6), all of them are qualitative (6, 7). Today, efforts have been made to quantify this criterion (8-10) and to determine standard questionnaires and diagnostic tools (11-13). In this way, the weight of each of the criteria should be specified, the diagnostic methods in each of the criteria should be carefully described (14), and then the resulting questionnaires should be checked for reliability and validity. So far, activities have been carried out in this field, the result of which is the production of some questionnaires (11, 12).

From the point of view of PM, a whole-body Mizaj is determined for every person based on the 10 criteria, which is a result of the Mizaj of the main organs, including the brain (15), liver (16), and heart (17). However, based on PM principles, determining the Mizaj in every age should be appropriate to that age range. So, using PM guidelines in the lifestyle of the elderly, determining the criteria for evaluating them from the perspective of Mizaj is important (18, 19). The Amirkola health and aging project (AHAP) is the first comprehensive cohort project of the elderly in Iran that started in 2011-2012 and composed 72.3% (1616 persons) of the population of elderly aged 60 years in Amirkola (20). Amirkola is a small town around Babol (one of the biggest cities in the North of Iran) with a middle-class income and low immigration rate. It can be an appropriate model of a city for cohort studies. More than 1,700 clinical and laboratory examinations of elderly people have been recorded. More than 50 articles were published in the first phase of AHAP)^21^(. The second phase of the evaluation of all the elderly (AHAP) in the city began in 2016-2017. This project was approved by the Research Ethics Committee of Babol University of Medical Sciences. All participants completed the informed consent form. Fifty-one medical professionals worked in this phase, a precious biobank was prepared, and more than 2000 clinical and laboratory examinations were obtained from more than 2000 elderly people in this phase. Trying to have an integration model for research in the field of traditional medicine, PM (also previously called Iranian traditional medicine) experts entered phase II of the project (21). As there was not any valid and reliable questionnaire for the elderly to evaluate Mizaj based on PM, the focus of PM experts was developing questionnaires based on quantitative indices to categorize the elderly. As defining a gold standard is necessary to develop questionnaires, a novel method of Mizaj assessment was introduced in this article.

**How did the evaluation of the Mizaj perform?**

All the elderly who entered the AHAP and were able to communicate verbally and consciously were assessed for Mizaj. This evaluation was performed in two ways:

1. Dr. Mojahedi’s questionnaire (12) was completed for all elderly. Considering that this questionnaire is the first questionnaire to determine whole-body Mizaj in middle-aged people, the evaluation of the elderly with this questionnaire had not been assessed by now. Therefore, the validation of this questionnaire in the elderly has been one of the objectives of the study.

2. Determining the Mizaj of the elderly by PM experts: Since there was no standard questionnaire in determining the Mizaj of the elderly based on PM, all the elderly were visited and their Mizaj was determined by one of 5 PM experts.

Mizaj was determined in two phases:

**First phase:** 5 to 10 elderly people were examined daily in the research center for AHAP every day. one PM expert (MD. PhD, professors of the school of Persian medicine, Babol University of Medical Sciences) attended the site and visited people, taking history and physical examination every day. Each visit lasted between 20 and 30 minutes, and videos and audio files were recorded from all stages of the visits. At this stage, a researcher-made checklist, containing 74 questions (based on PM references) was fulfilled (Table 1) and then the Mizaj of the whole body, heart, and brain, in two areas of warmness- coldness, and wetness-dryness by the experts were determined. Finally, at the end of each day, the elderly with a typical diagnosis of whole body Mizaj was marked. Although the 74-question-checklist was completed for each person, the diagnosis did have not a specific criterion and was determined based on the clinical experience of the PM experts. Typical (complete or partial) diagnoses were considered when the visiting expert sensed that the diagnosis of a person will likely be less controversial in the expert panel. At the end of this stage, a total number of 1636 elderly people were visited, out of them, 79 were identified as completely typical and 189 as partially typical (a total of 268 elderly). The files of these 268 elderly people were evaluated in the second stage (expert panel sessions).

**Second phase:** In this stage, the files of the elderly who were examined and visited in the first stage and whose diagnosis was considered typical were evaluated in an expert panel with the presence of 5 experts under the supervision of a methodologist. During this phase, an average of 30 to 45 minutes was spent on each elderly person. 

This phase was done in 3 steps:

Step 1: The expert who visited the elderly person, introduced the person using a sheet that recorded the patient's history, without stating his/her diagnosis.

Step 2: The audio file and the video taken by the examiner were played on a TV set. Based on the biography announced by the attending expert and the video seen, all 5 present experts, without any discussion, recorded their diagnosis secretly and separately on Mizaj of the whole body, heart, brain, and liver. They were recorded in two areas of Mizaj with numbers (1: warm, 2: moderate, 3: cold in the field warmness-coldness, and 1: wet, 2: moderate, 3: dry in the field of wetness-dryness). Step 3: These numbers were then announced by experts and recorded by the methodologist in a table. In cases where at least 4 out of 5 people made a diagnosis, the *complete agreement* was considered. In cases where 3 physicians made a diagnosis and the other 2 did not make counter-diagnoses, the *relative agreement* was considered. Cases that did not meet these conditions were considered disagreements. The basis for the presence or absence of agreement was the whole body Mizaj of the elderly. The details are in fig 1. At the end of this stage, 206 people had a complete agreement, 60 people had a partial agreement and 2 people disagreed with the whole body Mizaj of the elderly in the field of warmness-coldness and 1 in the field of wetness-dryness. The general results of Mizaj assessment are shown in table 1.

**Figure 1:**
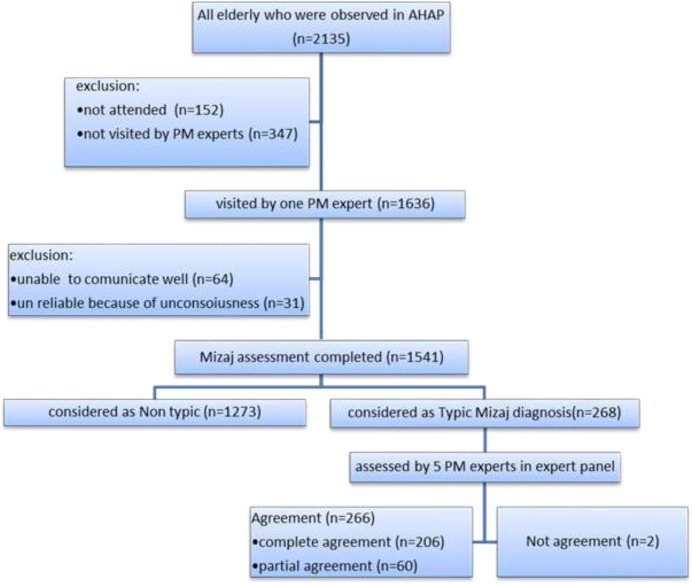
The flowchart of Mizaj assessment process in AHAP

**Table 1:** Details of final Mizaj assessment results in AHAP

		**Whole population** **(n=1541)**	**Expert panel** **(n=268)**	**Wetness-dryness**		**Whole population** **(n=1541)**	**Expert panel** **(n=268)**
Whole body Mizaj	Cold	315	69	Whole body Mizaj	wet	464	90
	Moderate	597	61		moderate	797	126
	Warm	629	136		dry	280	51
	Disagreement	-	2		Disagreement	-	1
	Missing	0	0		Missing	0	0
Heart	Cold	340	66	Heart	wet	346	48
	Moderate	512	61		moderate	731	153
	Warm	605	130		dry	366	52
	Disagreement	-	11		Disagreement	-	15
	Missing	84	0		Missing	98	0
Brain	Cold	250	51	Brain	wet	283	46
	Moderate	645	94		moderate	785	174
	Warm	561	113		dry	381	33
	Disagreement	-	10		Disagreement	-	15
	Missing	85	0		Missing	92	0
Liver	Cold	211	59	Liver	wet	516	108
	moderate	687	97		moderate	690	101
	Warm	457	103		dry	240	51
	Disagreement	-	9		Disagreement	-	8
	Missing	186	0		Missing	95	0

**How will the data of Mizaj be analyzed?**

The relationship between the results of assessing the whole body Mizaj as well as the Mizaj of the heart, brain, and liver with other laboratory and diagnostic data, quantitative criteria and standard questionnaires in AHAP will be determined. It will also be possible to provide statistical modeling to determine a diagnostic formula for Mizaj using quantitative indices. The information obtained in the whole body Mizaj will be analyzed at three levels:

1- Diagnosis with low-level reliability: The results of Mizaj assessment of 1541 elderly people. Each of them was visited at least by one expert. 

2- Diagnosis with moderate-level reliability: The sum of complete and relative agreement at the end of the second stage of determining the temperament, the Mizaj which includes 266 people.

3- Diagnosis with high-level reliability: Cases of complete agreement on determining the Mizaj, which reached 206 elderly people after two stages of work.

In all surveys, high-reliability data (n=206) will be the first step of the analysis, in which statistical modeling will be performed to see the relationship between each data and the final diagnosis. To control variations and increase the accuracy of coefficients, multilevel models with moderate effect (n=266) and low level (n=1541) will be analyzed by controlling the effect of the expert’s diagnosis and individual effects on the elderly.

**What are the main strengths and weaknesses?**

The main weakness of this study was the lack of visits of all the elderly by 5 PM experts. Since the visits of the elderly took about 18 months and it took between 4 and 5 hours every day, 5 experts of our team could not be present at the same time. To compensate for this weakness, a detailed written history, as well as audio and video recordings of all the steps of Mizaj assessment, was performed, and then in cases where it was diagnosed as typical, they were assessed in expert panel. A high sample size is one of the strengths of this study that could help to have enough samples after two phases of the study.

The presence of more than 2,000 clinical and laboratory data makes it possible to examine the relationship between Mizaj and each of these cases. This study is the first survey to determine the criteria for diagnosing Mizaj in the elderly based on PM. As the world is facing the phenomenon of aging and also as the criteria for assessing Mizaj are different in different ages, evaluating the elderly from this perspective,can help improve health in old age.

**Can I get hold of the data? Where can I find more information?**

Researchers can apply to access the data or suggest a collaboration study by submitting the proposal to the Traditional medicine and History of Medical Sciences Research Center, Babol, Iran. For more information, please contact the corresponding author. 
